# Detection and antibiotic resistance of *Mycoplasma gallisepticum* and *Mycoplasma synoviae* among chicken flocks in Egypt

**DOI:** 10.14202/vetworld.2020.1410-1416

**Published:** 2020-07-23

**Authors:** Marwa Emam, Yousreya Mohamed Hashem, Mahmoud El-Hariri, Jakeen El-Jakee

**Affiliations:** 1 VACSERA Holding Company for Biological Products and Vaccines, Cairo, Egypt; 2 Department of Mycoplasma, Animal Health Research Institute, Agriculture Research Center, Giza, Egypt; 3 Department of Microbiology, Faculty of Veterinary Medicine, Cairo University, Giza, Egypt

**Keywords:** *gapA* gene, *mgc2* gene, minimum inhibitory concentration, *Mycoplasma* infection, sodium dodecyl sulfate, *vlhA* gene

## Abstract

**Background and Aim:**

*Mycoplasma gallisepticum* (MG) and *Mycoplasma synoviae* (MS) are the most significant pathogens of avian mycoplasmosis. This study aimed to isolate and identify MG and MS from chickens and detect the various virulence genes in the isolates. Moreover, the efficacies of different antibiotics were tested to identify suitable treatment regimens.

**Materials and Methods:**

We isolated MG and MS from 487 chicken samples of different ages located in different Governorates in Egypt using conventional isolation methods. The isolates were characterized by polymerase chain reaction (PCR) and sodium dodecyl sulfate-polyacrylamide gel electrophoresis (SDS-PAGE) and then tested for antibiotic sensitivity by the minimum inhibitory concentration (MIC) method.

**Results:**

The prevalence of MG among the isolates was 9.85%, with the highest percentage isolated from air sacs, while the prevalence of MS among the isolates was 1.6%. Moreover, the highest levels of the prevalence of both MG and MS were during the winter and autumn sampling, while the lowest levels were in the summer and spring. Following the 16S rRNA-based detection of *Mycoplasma* isolates, 14 MG and 5 MS isolates were identified by different PCR-based detection methods for various virulence genes. Nine MG isolates contain the *mgc2* gene, six MG isolates contain the *gapA* gene, and three MS isolates contain the *vlhA* gene. We validated a duplex PCR method for the simultaneous identification of MG and MS, based on 100% of the MG and MS isolates generating common bands at 55 and 17 kDa, respectively. The MIC method identified tiamulin and spiramycin as the antibiotics of choice for the treatment of MG and MS infections, respectively.

**Conclusion:**

For more precise diagnosis of *Mycoplasm*a infections in chicken flocks, conventional isolation methods must be confirmed by PCR. SDS-PAGE analysis helps in epidemiological studies and vaccine preparation. The MIC method can be used to help develop therapies to control avian mycoplasmosis infections.

## Introduction

Mycoplasmas are small-celled prokaryotes belonging to the class *Mollicutes*. They have a small genome and are unable to produce a cell wall, as well as many metabolic pathways [1]. *Mycoplasma gallisepticum* (MG) and *Mycoplasma synoviae* (MS) cause the most significant *Mycoplasma* infections among commercial poultry. MG infections usually lead to chronic respiratory disease (CRD) in chickens and infectious sinusitis in turkeys. MS causes subclinical upper respiratory infections, eggshell apex abnormalities, and tenosynovitis or bursitis in chickens and turkeys [2-4]. CRD caused by MG is prevalent in layer, broiler, and breeder poultry flocks. Infected birds display respiratory symptoms that include sneezing, rales, coughing, as well as an exudation from the nostrils and eyes [5]. The presence of MG and MS in infected tissues or swab samples is confirmed by isolating the organisms using a specific medium or by direct detection of their DNA.

There are several types of respiratory infections that impose a significant health and economic impacts. For example, viruses and bacteria are known to coinfect chickens, although the pathogenesis involved, and their connection with mucosal surfaces are poorly described [6]. MG and MS diseases in chickens may sometimes superficially appear like respiratory diseases caused by other pathogens such as those that cause mild Newcastle disease and avian infectious bronchitis. Ishfaq *et al*. [7] found that MG causes CRD in chickens, and infected chickens display abnormal morphology and cellular damage, including increases in inflammatory cell infiltration, cellular debris, and exudates, and mitochondrial and DNA damage in lungs. Infections with *Avibacterium paragallinarum* and *Pasteurella multocida* may present similarly and should be excluded. MG in turkeys may be confused with avian pneumovirus infections, and the presence of sinusitis may also be suggestive of infections by *P. multocida*, *Chlamydia*, or MS. Moreover, care should be exercised in distinguishing between MS-related infectious synovitis, *Staphylococcus aureus* infections, and reovirus-related infectious tenosynovitis [8]. In general, MG and MS cause significant impacts on the poultry industry, thus detecting these pathogens is crucial for conducting epidemiological studies and control programs of avian mycoplasmosis.

This study aimed to characterize MG and MS isolates from chicken flocks using molecular methods and to test these isolates for antibiotic sensitivity by the minimum inhibitory concentration (MIC) method, thus providing a guide to optimal therapy.

## Materials and Methods

### Ethical approval

The study protocol was approved by Animal Ethics Committee of the Faculty of Veterinary Medicine, Cairo University, Egypt.

### Sampling

We collected 487 chicken samples from different flocks, breeds, and ages in Egypt (Giza, Fayoum, Benisuef, Menya, and Alexandria) from January 2017 to July 2019.

Samples were from healthy (n=50), morbid (n=133), and freshly dead (n=304) birds suffering from respiratory and locomotor problems.

In acute cases, tracheal swabs, synovial fluid, trachea and lung samples were collected; while in chronic stages, air sac specimens and caseous material were collected (Table-1).

**Table-1:** Type and number of the examined samples.

Type of sample	Healthy birds	Morbid birds	Freshly dead birds	Total
Tracheal swab	50	133	-	183
Trachea	-	-	63	63
Lung	-	-	118	118
Air sacs	-	-	51	51
Caseous material	-	-	2	2
Synovial fluid	-	-	70	70
Total number	50	133	304	487

### Culturing, isolation, and preliminary identification

The isolation and preliminary identification of *Mycoplasma* isolates followed the standard procedures defined by Kleven [9]. The samples were cultured on sterile PPLO broth and agar media, as described by Frey [10]. *Mycoplasma* colonies were identified based on their characteristic fried egg appearance [11].

The digitonin sensitivity test was performed to differentiate between *Mycoplasma* and *Acholeplasm*a isolates [12]. The glucose fermentation test [13] and the arginine hydrolysis test [14] were performed to differentiate Avian *Mycoplasma* species. Finally, the film and spot formation test used to identify *Mycoplasma synoviae* [15] and the growth inhibition test [16] were performed to identify a *Mycoplasma* isolate depending on serology.

### Purification and maintenance of isolates [17]

A single fried egg-shaped colony was picked, including the agar block, and transplanted into a broth medium to obtain a pure culture. The purified isolates were kept at −20°C in the form of agar blocks.

### Sodium dodecyl sulfate-polyacrylamide gel electrophoresis (SDS-PAGE)

The isolates were characterized to determine strain variability. SDS-PAGE followed the methods described by Laemmli [18]. The samples were run alongside preserved molecular weight markers ladder from Cleaver Scientific Ltd (Blue Wide Range Prestained Protein Ladder, UK) and low molecular weight protein ladder (Pharmacia Biotech, USA). 200 μl of activated MG or MS Broth culture was mixed with equal volume of denaturation mixture containing 2-mercaptoethanol. The mixture was boiled for 5 min then 20 μl was applied to each well of the SDS-PAGE gel. The gel was run at a constant power of 25 mA for approximately 4-5 h using a horizontal electrophoresis connected with power supply (Biometra, USA). Low molecular weight and wide range protein ladders were included in each run. The gel was stained overnight (50% methanol, 7% glacial acetic acid, and 0.2% Coomassie Brilliant Blue) and de-stained using a solution containing 40% methanol and 7% glacial acetic acid. The bands were visualized, photographed, and analyzed using Alpha View-AlphaImager HP (ProteinSimple, CA, USA).

### Polymerase chain reaction (PCR)

DNA of each isolate was extracted using the Quick-DNA™ Universal Kit catalog no D4068 and D4069 (Qiagen, USA. PCR was conducted using specific primers and amplification cycles according to the references as shown in Table-2 [8,19-22]. Aliquots (10 μl) from each reaction mixture were analyzed by electrophoresis (1.5 volts, 45 min) using 1.5% (weight/volume) agarose in 1 × TBE (Tris, boric acid, EDTA, pH 8.0). After electrophoresis, gels were stained with ethidium bromide (1 μl/ gel) and visualized and photographed with an ultraviolet transilluminator and camera system [23].

**Table-2:** Primers used for PCR analysis.

Microorganism	Gene	Primer 5’- 3’	Amplicon size	Reference
*Mycoplasma gallisepticum*	16S rRNA	F-GAG-CTA-ATC-TGT-AAA-GTT-GGT-C R-GCT-TCC-TTG-CGG-TTA-GCA-AC	185 bp	[8]
	*mgc*2	F-CGCAATTTGGTCCTAATCCCCAACA R-TAAACCCACCTCCAGCTTTATTTCC	300 bp	[20]
	*gap*A	F-GCCGGA TTG ATT TGT ATG R-ACT TGT TTT GTG TTT CC	1511 bp	[21]
*Mycoplasma synoviae*	16S rRNA	F-GAG-AAG-CAA-AAT-AGT-GAT-ATC-A R-CAG-TCG-TCT-CCG-AAG-TTA-ACA-A-	207 bp	[8]
	*Vlh*A	F-GTACGGTGTTAAGTCATC R-CGTATTTACAGCACCAGTAGTAACT	1100bp	[22]
Both (Duplex)	MG: IGSR (16S-23S rRNA)	F-GTAGGGCCGGTGATTGGAGTTA R-CCCGTAGCATTTCGCAGGAGTTA	812bp	[23]
	MS:16S rRNA	F-GAGAAGCAAAATAGTGATATCA R-CAGTCGTCGTCTCCGAAGTTAACAA	207bp	[8]

PCR: Polymerase chain reaction

### Microbroth antibiotic sensitivity testing

MG and MS isolates were treated with a range of concentrations(64 μg/ml-0.0625 μg/ml) of the following antibiotics: Ciprofloxacin 100 mg/ml (Agrovetica Egypt), doxycycline 100 mg/ml (Primavet, Egypt), linco spectinomycin (lincomycin 50 mg/ml and spectinomycin 100 mg/ml) (Delta Pharma, Egypt), oxytetracycline 30 mg/ml (Pfizer, Egypt), spiramycin 150,000,000 IU (Merial, France), tiamulin 450 mg/g (Novartis, Switzerland), tilmicosin 25% (Atco Pharma, Egypt), and tylosin 200 mg base/ml (Adwya, Egypt). The experiment was designed to determine the lowest concentration that completely prevents microbial growth, as described by Hannan [24].

## Results

### Occurrence of *Mycoplasma* isolates in the samples

Table-3 shows that MG was isolated from 9.85% of the samples consisting of tracheal swabs, trachea, lung, air sacs, and caseous materials. Meanwhile, MS was isolated only from the synovial fluid (1.6%). The results clearly show that the highest incidence of *Mycoplasma* infections occurs during winter.

**Table-3:** *Mycoplasma gallisepticum* and *Mycoplasma synoviae* isolated from live and freshly dead birds with reference to seasonal prevalence.

Type of sample	Number of the examined samples	MG					MS				
	
		Positive number (%)				Total number of positive (%)	Positive number (%)				Total number of positive (%)
	
		Winter	Autumn	Summer	Spring		Winter	Autumn	Summer	Spring	
Tracheal swabs*	183	9 (4.9)	3 (1.63)	1 (0.5)	1 (0.5)	14 (7.7)	-	-	-	-	-
Trachea	63	3 (4.7)	3 (4.7)	1 (1.58)	0	7 (11.11)	-	-	-	-	-
Lung	118	10 (8.47)	6 (5.08)	1 (0.85)	0	17 (14.4)	-	-	-	-	-
Air sacs	51	4 (7.8)	3 (5.88)	1 (1.96)	0	8 (15.7)	-	-	-	-	-
Caseous material	2	2 (100)	0	0	0	2 (100)	-	-	-	-	-
Synovial fluid	70	0	0	0	0	-(0)	2 (2.85)	2 (2.85)	1 (1.4)	0 (0)	5 (7.1)
Total	487	28 (5.74)	15 (3.08)	4 (0.82)	1 (0.2)	48 (9.85)	2 (0.4)	2 (0.4)	1 (0.2)	0 (0)	5 (1.6)

* Tracheal swab used in isolation of *Mycoplasma gallisepticum* only from 50 healthy and 133 morbid birds, no positive samples detected in healthy birds and 14 positive samples detected in morbid birds

### SDS-PAGE

**Figure-1:**
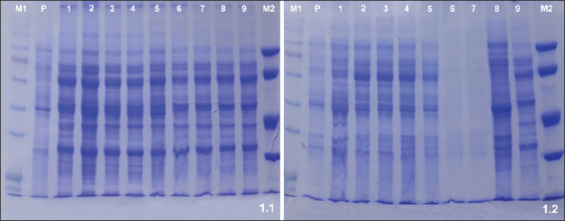
Sodium dodecyl sulfate (SDS)-polyacrylamide gel electrophoresis among *Mycoplasma* isolates. 1.1): SDS-Polyacrylamide gel electrophoresis among MG isolates. Lane M1: A high molecular weight marker, lane P: Reference strain, lanes 1-9: *Mycoplasma gallisepticum* isolates and Lane M2: Low molecular weight marker.1.2): SDS-polyacrylamide gel electrophoresis among MS isolates. Lane M1: High molecular weight marker. Lane P: Reference strain, lanes 1-9: *Mycoplasma synoviae* isolates (Lane 1 and 9 [first MS isolate] Lane 2 and 3 [second MS isolate] Lane 4 and 5 [Third MS isolate] Lane 6 and 7 [fourth MS isolate] Lane 8 [fifth MS isolate]) and Lane M2: Low molecular weight markers.

The MG isolates; 60% (5/9 isolates) shared bands at 73, 72, 56, 53, 47, 46, and 24 kDa; 70% (6/9 isolates) shared bands at 54 and 20 kDa; 80% (7/9 isolates) shared a 70 kDa band; 90% (8/9 isolates) shared a 19 kDa band; and all isolates shared a 55 kDa band (Figure-1:1.1).

The MS isolates; 60% (3/5 isolates) shared bands at 72, 30, 27, 25, and 22 kDa; 80% (4/5 isolates) shared 37 and 20 kDa bands; and all of the isolates shared a 17 kDa band (Figure-1).

### PCR

In PCR analysis of 16 *Mycoplasma* isolates was analyzed by PCR to specifically amplify the 16S rRNA gene. Only 14 isolates were identified as MG, while the *mgc2* and *gapA* virulence gene was detected in only nine and six of the 14 MG isolates, respectively.

Five *Mycoplasma* isolates were analyzed by PCR. Based on their 16S rRNA gene, all five isolates are classified as MS. The *vlhA* virulence gene was detected in only three out of five MS isolates.

**Figure-2:**
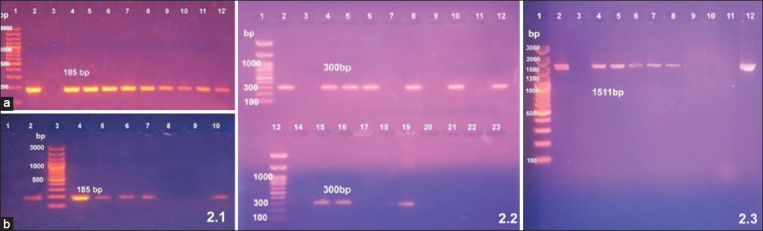
Results for polymerase chain reaction (PCR) for the detection of 16S rRNA, *mgc2*, and *gapA* genes among the isolates. 2.1): Agarose gel electrophoresis of amplified PCR products of 16S rRNA gene among *Mycoplasma gallisepticum* at 185 bp. (a): Lane 1: 100 bp DNA marker (Thermo Fisher Scientific, USA), lane 2: Positive control, lane 3: Negative control, lanes: 4-12: MG positive isolates. (b): Lane 1: Negative control, lane 2: Positive control, lane 3:100 bp DNA marker (Thermo Fisher Scientific, USA), lanes 4-7 and 10: MG positive isolates. Lanes 8 and 9: Negative *M. gallisepticum* isolates. 2.2): Agarose gel electrophoresis of amplified PCR products of *mgc2* gene of *M. gallisepticum* isolates at 300 bp. Lanes 1 and 13: 100 bp DNA marker (Gene Direx, USA), Lane 2: Positive control, Lanes 3 and 14: Negative control, Lanes 4, 5, 6, 8, 10, 12, 15, 16, and 19: Positive *M. gallisepticum* (*mgc2* gene) isolates and lanes 7, 9, 11, 17, and 18: Negative *M. gallisepticum* (*mgc2* gene) isolates. 2.3): Agarose gel electrophoresis of amplified – PCR products of *gapA* gene of *M. gallisepticum* isolates at 1511 bp. Lane 1: 100 bp DNA marker (Thermo Fisher Scientific, USA), Lane 2: Positive control, Lane 3: Negative control, Lanes 4, 5, 6, 7, 8, and 12: Positive *M. gallisepticum* (*gapA* gene) isolates and lanes 9, 10, and 11: Negative *M. gallisepticum* (*gapA* gene) isolates.

**Figure-3:**
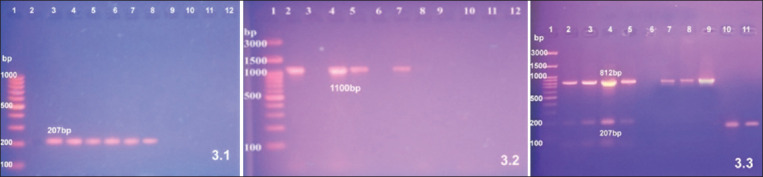
Results for polymerase chain reaction (PCR) for the detection of 16S rRNA, *vlhA*, and 16S-23S rRNA genes among the isolates. 3.1): Agarose gel electrophoresis of amplified PCR products of rRNA16S rRNA gene of among *Mycoplasma synoviae* isolates at 207 bp. Lane 1: 100 bp DNA marker (Thermo Fisher Scientific, USA), lane 2: Negative control, lane 3: Positive control, lanes 4 to 8: Positive MS isolates. 3.2): Agarose gel electrophoresis of amplified PCR products among *vlhA* gene of *M. synoviae* isolates at 1100 bp. Lane 1: 100 bp DNA marker (Gene Direx), Lane 2: Positive control, Lane 3: Negative control, Lanes 4, 5 and 7: Positive *M. synoviae* (*vlhA* gene) isolates and Lanes 6 and 8: Negative *M. synoviae* (*vlhA* gene) isolates. 3.3): Agarose gel electrophoresis of amplified PCR products of both 16S-23S rRNA gene among *Mycoplasma gallisepticum* (MG) and *M. synoviae*. Lane 1: 100 bp DNA marker (Thermo Fisher Scientific, USA), Lanes 2-4: Showing amplification of 812 and 207 bp products of MG and MS, respectively, Lane 5: Positive MS strain Lane 6: Negative control, Lanes 7-9: Showing amplification of 812 bp product of MG, Lanes 10 and 11: Showing amplification n of 207 bp product of MS.

Duplex PCR analysis was designed to simultaneously detect MG and MS by targeting the *16S*-*23S* rRNA gene region of the isolates. MG positive results were 14/16 (87.5%). 14 MG OIE positive isolates were tested for virulence using *mgc2* virulent gene and *gapA* virulent gene and we found that 9/14 were *mgc2* positive isolates (64.3%) and 6/14 were *gapA* positive isolates (42.9%) (Figure-2). MS positive results were 5/5 (100%). MS OIE positive isolates were tested for virulence using *vlhA* virulent and we found 3/5 were *vlhA* gene-positive isolates (60%) (Figure-3). Results indicated a difference in virulence between the isolates. Finally, we adopted and validated duplex PCR for simultaneous identification of MG and MS as it was more rapid and cheaper method in comparison to single PCR for identification of MG and MS.

###  Microbroth antibiotic sensitivity test

Antimicrobial sensitivity tests were conducted on 5 MG and 3 MS isolates using the MIC method (Table-4). The MIC values among the isolates ranged from 0.0625 to 0.5 ug/ml.

**Table-4:** Microbroth antibiotic sensitivity test (minimum inhibitory concentration).

Antimicrobial	Isolates (MG)					Range	Isolates (MS)			Range
Ciprofloxacin	0.0625	0.0625	0.125	0.0625	0.0625	0.0625-0.125	0.0625	0.0625	0.125	0.0625-0.125
Doxycycline	0.25	0.0625	0.125	0.0625	0.0625	0.0625-0.25	0.5	0.125	0.125	0.125-0.5
Lincospectin mycin	0.0625	0.25	0.125	0.0625	0.0625	0.0625-0.25	0.25	0.125	0.125	0.125-0.25
Oxytetracycline	0.0625	0.25	0.25	0.0625	0.125	0.0625-0.25	0.0625	0.125	0.125	0.0625- 0.125
Spiramycin	0.0625	0.25	0.25	0.125	0.125	0.0625-0.25	0.0625	0.0625	0.0625	0.0625
Tiamulin	0.0625	0.0625	0.0625	0.0625	0.0625	0.0625	0.5	0.25	0.0625	0.0625-0.5
Tilmicosin	0.0625	0.125	0.5	0.0625	0.0625	0.0625-0.5	0.25	0.125	0.0625	0.0625-0.25
Tylosin	0.0625	0.5	0.25	0.125	0.0625	0.0625-0.5	0.5	0.125	0.0625	0.0625-0.5

MS=*Mycoplasma synoviae*, MG=*Mycoplasma gallisepticum*

## Discussion

MG and MS are important pathogens that affect poultry worldwide, causing enormous economic losses in the poultry industry [25,26].

MG is a major poultry pathogen [27] that causes CRD in chickens and turkeys and also negatively impacts egg hatchability and development [28]. MG infections also affect virus titers and the efficacy of some poultry viral vaccines [29,30]. In our study, analysis of tracheal swabs, trachea, lungs, and air sac samples showed that MG is most prevalent in the air sacs (15.7%), a result that is similar to that reported by Reda and El-Samie [31] and and Mohamed [32], who isolated MG from air sacs at the highest rates of 23.3% and 21.5% respectively. Meanwhile, EI-Jakee *et al*. [33] isolated and cultured 14 *Mycoplasma* isolate from tracheal swabs collected from 12 broiler breeder flocks.

Necropsy of the diseased birds revealed chronic macroscopic lesions accumulating as caseous material in the lungs [34]. This material is sticky, attaching to the respiratory surface of the air sacs. All of the MG isolates in our study were obtained from caseous material.

MS is an important poultry pathogen that causes air sacculitis, synovitis, and reduced egg production, resulting in great economic losses [4,35]. The prevalence of MS in the synovial fluid was only 7.1%. This low percentage is attributed to the difficulty in isolating MS due to its fastidious growth.

MG and MS were digitonin positive, glucose fermentation positive, and arginine hydrolysis negative. Meanwhile, only suspected MS isolates gave positive results in the film and spot assay. In the growth inhibition test, 85% of suspected MG isolates were inhibited by specific antiserum against MG, while all MS isolates were inhibited by anti-MS antiserum. These results agree well with the results of biochemical identification.

The present study shows that MG and MS infections are more predominant during winter and autumn compared to summer and spring. This seasonal difference is probably due to poor temperature control measures, resulting in low-temperature stress [36].

SDS-PAGE has been used to demonstrate minor but distinct and reproducible differences in protein patterns among strains of MG and MS. Therefore, SDS-PAGE is a useful procedure in epidemiological and other studies, where it is important to be able to distinguish between bacterial strains based on protein patterns. The molecular detection of pathogenic isolates of MG by SDS-PAGE was indicated by the presence of specific 72 and 73 kDa bands in 60% of the isolates, which was also observed by Hassan *et al*. [36].

Strategies to control MG and MS infections are based primarily on serological and/or bacteriological screening. However, culture-based methods are laborious, time-consuming and may fail to detect *Mycoplasma* species from medicated birds. They are also less sensitive than PCR-based methods. PCR provides a rapid, sensitive, and precise method for diagnosing MG from suspected cases [5].

MG and MS isolated from chicken flocks were biochemically tested and then identified using PCR. Targeting using the 16S rRNA gene, PCR positively identified 87.5% and 100% of the MG and MS isolates, respectively. This finding agrees with those of Atique *et al*. [37] who confirmed that PCR provides more reliable, specific, and rapid results compared to culture.

The isolates that were positive for the *mgc2* and *gapA* genes accounted for 64.3% and 42.9% of the MG OIE-positive isolates. The isolates that were positive for the *vlh* gene accounted for 60% of the MS OIE-positive isolates. These results suggest that the isolates may differ in their respective virulence levels. Therefore, Norouzian *et al*. [38] highlighted the importance of understanding the relationship between changes in MG antigenicity and pathogenicity to genetic variations.

Duplex PCR is cheaper and faster than single PCR for the identification of MG and MS. It is, therefore a good alternative method. The duplex technique was able to detect MG and MS simultaneously, although the MG bands are more intense than those of MS [39]. In our study, we adopted and validated duplex PCR for the simultaneous identification of MG and MS.

The PCR technique not only has several advantages, but it is also limited by the development of severe contamination due to the improper handling of samples, thus resulting in false-positive results [40,41]. Therefore, conventional cultural methods should be carried out in parallel with PCR.

Abd El-Hamid *et al*. [42] demonstrated that MG and MS infections can be controlled by proper treatment procedures. To develop an effective antibiotic treatment for MG and MS infections, we performed MIC experiments using eight different antimicrobials against five and three different MG and MS field isolates, respectively. On the one hand, tiamulin was the most effective antimicrobial for inhibiting MG growth *in vitro*. Thus, we recommend treating MG-infected birds using this antimicrobial, which agrees with Xiao *et al*. [43], who concluded that tiamulin is highly active against MG. On the other hand, spiramycin was the most effective antimicrobial for inhibiting the growth of MS *in vitro*. The use of this antibiotic has not yet resulted in any case of mycoplasma resistance; thus, we recommend it for treating MS infection. Hong *et al*. [44] suggested treating MS infections with antibiotics that do not cause the rapid development of MS resistance.

## Conclusion

MG and MS are the predominant species of *Mycoplasma* that causes respiratory and joint problems in chickens in the different Egyptian governorates (Giza, Fayoum, Benisuef, Menya, and Alexandria). Although time-consuming, the traditional cultural approach remains the gold standard for MG and MS diagnosis. PCR-based methods are more specific and faster than traditional culture-based methods; however, they should be performed in parallel with the traditional culture-based method. Periodic *in vitro* testing of MICs of antibiotics against *Mycoplasma* field isolates is required to monitor the impact of mass medication programs and to assist in developing effective therapies.

## Authors’ Contributions

ME collected the data, participated in the design of the work protocol, performed the laboratory work, and was involved in the preparation of the manuscript. ME and YMH found the research idea, shared in the performed data and designed the work protocol. JE participated in the design of the work protocol, contributed to the manuscript review and interpreted the results. All authors were involved in the preparation of the manuscript and had read and approved the final manuscript.
